# Victimization in Youth with Autism Spectrum Disorder in Tunisia: Prevalence and Associated Factors

**DOI:** 10.1192/j.eurpsy.2025.844

**Published:** 2025-08-26

**Authors:** R. Ayoub, A. Beji, H. Ben Abid, K. Younes, A. Guedria

**Affiliations:** 1 Child and Adolescent Psychiatry Department, Fattouma Bourguiba university Hospital-University of Monastir; 2 Regional Rehabilitation Unit, Monastir, Tunisia

## Abstract

**Introduction:**

Autism Spectrum Disorder (ASD) is a neurodevelopmental disorder that attracts interest, fascination, and controversy. Due to their developmental characteristics that hinder autonomy and socialization, children with ASD appear to be vulnerable and prone to victimization. Indeed, victimization has severe consequences for these children, who face risks of rejection and stigmatization. However, research on this phenomenon in Tunisia is still limited, highlighting a significant gap in the current knowledge base.

**Objectives:**

Our study aimed to determine the prevalence of victimization among children and adolescents with ASD consulting at the Regional Rehabilitation Unit of Monastir- Tunisia and identify the associated factors.

**Methods:**

This is a cross-sectional analytical study. It included children and adolescents with ASD aged between 5 and 18 years consulting at the Regional Rehabilitation Unit in Monastir during the year 2023. Sociodemographic and clinical data of the children were collected using a pre-established form created by the working group. We used the Juvenile Victimization Questionnaire ScreenerSumVersion (JVQ-SSV) to assesse childhood victimization and major forms of aggression against youth.

**Results:**

Our study included 74 participants, demonstrating a male predominance with a sex ratio of 6.4. The average age of the patients was 9 years, ranging from 6 to 14 years.The main results of our study showed that participants with ASD are particularly vulnerable to victimization, with 77% having experienced at least one victimizing event in their lifetime and nearly 69% in the past year. The most commonly reported victimization events included bullying (56.7%), peer or sibling aggression (50%)… Our results revealed that 56.2% of these incidents occurred at school. Additionally, nearly 46% of children with ASD were reported by their parents to be perpetrators of victimization towards other. Univariate analysis identified significant factors associated with victimization, including the parent’s age, attendance at specialized education centers, and being a perpetrator of victimization.

**Conclusions:**

The social inclusion of children with ASD, though crucial, remains fragile amid widespread victimization in both school and family environments. The concerning repercussions of these experiences necessitate focused efforts to develop and implement strategies that safeguard and support these children. Addressing the multifaceted nature of victimization and promoting protective measures are essential for improving their well-being and fostering a more inclusive environment.

**Disclosure of Interest:**

None Declared

